# The Evaluation Simulator: A New Approach to Training Music Performance Assessment

**DOI:** 10.3389/fpsyg.2019.00557

**Published:** 2019-04-09

**Authors:** George Waddell, Rosie Perkins, Aaron Williamon

**Affiliations:** ^1^Centre for Performance Science, Royal College of Music, London, United Kingdom; ^2^Faculty of Medicine, Imperial College London, London, United Kingdom

**Keywords:** evaluation, simulation, assessment, performance, immersive virtual environment (IVE)

## Abstract

A growing body of work has examined the act of evaluating the quality of a musical performance. This article considers the domain of training evaluative skills in musicians, presenting assessment as a form of performance to be taught and demonstrating a gap in opportunities for trainees to develop evaluative skills within the heightened environments of live assessment scenarios. To address these needs, the concepts of Immersive Virtual Environments (IVEs) and distributed simulation are described, highlighting their use in training and research in other performance domains. Taking this model as a starting point, we present the *Evaluation Simulator* as a new tool to study and train performance evaluation. Potential applications of this prototype technology in pedagogical and research settings are then discussed.

## Introduction

Evaluation is surely a skill.^1^ Good evaluations can be defined, and good evaluators distinguished. At least, this is the assumption on which any formal assessment scheme incorporating an “expert” assessor is based (Thompson and Williamon, 2003). However, the concept of the skillful, professional evaluator is not one to be taken for granted. A great deal of study has examined the products and processes of forming music performance quality evaluations (see McPherson and Schubert, 2004; Waddell and Williamon, 2017a for reviews) Despite the crucial role such assessments play in the development and careers of musicians, research has demonstrated a worrying degree of variability and subjectivity (Thompson and Williamon, 2003), including the influence of extra-musical visual factors (Elliott, 1995; Griffiths, 2008, 2010; Platz and Kopiez, 2013; Waddell and Williamon, 2017b), issues of rater consistency and inter-rater reliability (Wesolowski et al., 2015, 2016), and a lack of standardization in the scales and rubrics used (Russell, 2015; Kopiez et al., 2017). Previous studies have questioned the value of an evaluator's expertise in delivering reliable and consistent judgments (e.g., Fiske, 1975, 1977; Winter, 1993). A review of 86 articles examining the abilities of music teachers in classroom or lesson settings found a high degree of variability in the nature and effectiveness of their feedback, even within a single lesson (Duke, 1999). This is all not to say that there does not exist an evaluative skill, or that such a skill is not valuable, but simply emphasizes the point that one's ability as a musical performer does not automatically translate to ability as an effective judge. Indeed, the profession of the instrumental music teacher (and, by extension, music examiner or competition judge) is populated primarily not by those with significant training in evaluation but rather by those who have demonstrated significant ability in the specialist area on which they are passing judgment, i.e., performance.

This is not due to lack of effort by those evaluators, or those who have assigned them. Rather, it indicates the lack of opportunities for this training, and the assumptions underlying what comprises an expert evaluator. The celebrated violinist Joseph Szigeti noted this in his autobiography, speaking of the challenges faced by the expert music judge and critic:

This comparison of performances (whether of those by the same player spread over a given length of time, or of performances of the same works by about equally qualified players, massed within a short period) should be one of the self-imposed tasks of all conscientious critics. I don't quite know how they could manage it; perhaps by attending contests, examinations, and the like, taking a kind of post-graduate course in performance-criticism. As far as my own experience goes, my duties as member of the jury at the Paris Conservatoire contests and at the Brussels Concours International provided me with invaluable object lessons in the field of critical listening. On an active practitioner such lessons are wasted, of course, whereas for a critic…. (Szigeti, 1947, p 254, ellipses in original).

In this context Szigeti is referring to the “critic” in the sense of a critical reviewer, one publishing written reports and reviews of public performances. However, the translation can be made to the evaluator, as critics must also deconstruct the salient aspects of the performance (e.g., technique, artistic style, control, interpretation, etc.), make comparisons across performances, and translate this to a form of feedback that provides a desired outcome for a particular audience/reader (Alessandri et al., 2014, 2015). With this in mind, Szigeti makes several salient points in the quotation. First, he addresses the challenge of making consistent and reliable comparisons between performances separated by time or between interpretations. The research literature has emphasized this difficulty, most notably in studies demonstrating how experienced listeners can often mistake the same performance played twice as two distinct interpretations (Duerksen, 1972; Anglada-Tort and Müllensiefen, 2017). Second, Szigeti struggles to identify a programme by which one could develop this skill, suggesting experience through exposure and a hypothetical course of advanced study, although seemingly unaware of whether such a programme or degree exists. Even if he is speaking of the specific skill of published performance criticism, a course on performance evaluation would seem to be a clear analog. He confirms this view in his third point, where he highlights his role as jury member for a number of internationally prominent panels as his own lessons in criticism. Thus, he learned to assess by undertaking the assessment of others, in the process contributing to decisions having considerable ramifications for those assessed without any specific education in how to conduct them. He concludes by suggesting that such lessons are wasted on an “active practitioner” (meaning performer?) but have value for the critic.

This quotation by a prominent musician from the relatively recent history of the Western classical tradition highlights the degree to which the skill of evaluation has been given far less attention than the skill of performance. It suggests that those in positions of evaluative power are chosen not for their ability as judges, but for their prominence in a related domain. Such a view would be in line with the history of skill assessment. Centuries earlier, the apprenticeship model of developing skilled crafts once favored social class in determining who held the power to assess and determine worth, a trend that shifted in 19th century Europe with the rise of competitive assessment, individualism, and a gradual (and unfinished) transition from a hierarchy based on class structure to one of meritocracy (Eggleston, 1991). It is notable, therefore, that the method of training modern musicians, at least those in the Western classical tradition, remains based largely upon the master-apprentice model (Gaunt, 2017). Conservatoires heavily favor the training of performance skills (Perkins, 2013), while the skill of performing effective evaluations receives far less attention. This is despite the fact that the ability to dissect and deliver useful feedback upon performance is central to the career of the modern portfolio musician, who is likely to have multiple roles as performer, assessor, and teacher (Bennett, 2008).

A few exceptions to this can be found. The Associated Board of the Royal Schools of Music (ABRSM), for instance, requires training, professional development, and monitoring for its 700 examiners through a 3-day introductory course and subsequent 4 days of sessions that emphasize learning through the conducting of mock or true evaluations under the guidance of those more experienced evaluators (Stewart, 2011). Examiners are also periodically moderated, during which a second examiner remains in the room for the full session. Such practices have also been piloted and employed in higher education settings, examples of which are discussed later, although the practice is not widespread.

The practice and skill of evaluation delivery has been given greater attention, at least in terms of research and discussion, in the domain of classroom-based and higher-education teaching. Goolsby (1999) defined four functions of assessment; (1) placement, in which performances are ranked or chosen; (2) summative, in which a performance evaluation is used to summarize ability or a period of learning; (3) diagnostic, used to pinpoint learning and technical deficiencies; and (4) formative, to determine whether development has taken place and to foster continued learning. Research and practice in evaluation in the wider educational context has focused on the third and fourth categories in their role in enhancing student learning and development. Nicol and Macfarlane-Dick (2006) identified seven principles of good practice in the delivery of formative assessment. They encouraged feedback that:

1. helps clarify what good performance is (goals, criteria, expected standards);
2. facilitates the development of self-assessment (reflection) in their learning;
3. delivers high quality information to students about their learning;
4. encourages teacher and peer dialogue around learning;
5. encourages positive motivational beliefs and self-esteem;
6. provides opportunities to close the gap between current and desired performance;
7. provides information to teachers that can be used to help shape teaching.

These principles share close ties with those of self-regulated learning, which theorizes that effective learning happens when learners deliberately plan, execute, and review their practice, working toward concrete goals while maintaining a metacognitive awareness that allows them to monitor and adapt their cycle of learning depending on their individual and subject-specific challenges (Zimmerman, 1990; Jørgensen, 2004, 2008). This can foster practice that is considered and deliberate, features critical to achieving peak performance outcomes (Ericsson et al., 1993). Paris and Winograd (1990) proposed that regular self-assessment of learning processes and outcomes promotes more effective monitoring of progress, facilitates the identification and correction of mistakes, and enhances feelings of self-efficacy, which is the belief in one's ability to perform domain-specific skills (Bandura, 1997; McCormick and McPherson, 2003; McPherson and McCormick, 2006; Ritchie and Williamon, 2011) and has been linked to improvements in practice (Ritchie and Williamon, 2012). Reciprocally, increased self-efficacy has been found to lead to higher self-evaluations, which themselves become increasingly underconfident as performance ability increases (Hewitt, 2015). In general, self-assessments are found to be higher than those of third-party experts (Hewitt, 2002, 2005). Such optimism in self-assessment has been linked to higher performance achievement and persistence in comparison with students displaying more realistic or pessimistic tendencies (Bonneville-Roussy et al., 2017). Effective feedback, especially feedback that motivates and facilitates self-assessment, allows learners to close the cycle of self-regulated learning and enhance their performance practice most effectively. If this practice is performing the skill of assessment, then one must learn to self-assess one's ability to assess.

This ability to self-regulate feedback delivery forms a subset of what Medland (2015) defines as assessment literacy. In a study of external examiners in UK higher education she found deficits across six categories: (1) *community*, or degree to which examiners had knowledge of and participated in groups sharing good practice; (2) *standards*, or the knowledge of and adherence to institutional and national policies; (3) *dialogue*, or the role and methods of engaging with students in their feedback and fostering peer-to-peer dialogue; (4) *self-regulation*, or the ability to demine and improve the quality of their own feedback; (5) *programme-wide approach*, or knowledge of and integration with the wider institutional and learning context for the material being taught and assessed, and (6) *knowledge and understanding*, or familiarity with the underlying pedagogical and psychological principles of effective assessment. Medland found a significant emphasis on *standards*, especially relating to the consistency, transparency, and appropriateness of the assessment policies in place. Such focus on procedure and policy invokes the danger of what Ferm Almqvist et al. (2016) defined as “deformative” assessments, where over-assessed learning can promote a culture of criteria compliance rather than individualized self-regulated learning practices. Emphasizing this, Medland found the category of *self-regulation* to be the least-mentioned component in her cohort. Responses relating to *dialogue* also highlighted an emphasis on one-directional feedback delivery rather than constructive and formative interaction between instructor and student or, indeed, between external examiners, programme leaders, and lecturers. The importance of the methods of feedback delivery should not be overlooked. Not only do they provide new opportunities for formative learning, but the assessor's style and language can have a greater effect on the students' perceived value of the criticism and resulting self-confidence than the pedagogical content itself (Bonshor, 2017). It is here that the “performance” of an effective evaluation is crucial.

### Evaluation as Performance

While performance evaluation can be conceptualized as a unique skill to be developed, there is value in considering it as an act of performance in itself. Like the musical performance it seeks to quantify, it calls upon specialist knowledge. It takes place in specific settings, often involving interaction with a team of familiar and/or unfamiliar experts that may or may not share a specific sub-specialism. It can take place in front of an audience (as in public competitions), one that can be critical of the outcome. The results of the act have consequences, not only for those being assessed, but for the evaluative performer in its effects on their reputation, standing, and employability as an evaluator. And, it is a process that unfolds in a fixed sequence over a fixed amount of time, often limiting or outright preventing opportunity for pause, repeat, or reflection, and including distinct periods of pre- and post-performance activities. To examine evaluation through the lens of performance allows us to consider its treatment anew. Evaluation is not just a tool to summarize, diagnose, and develop performance; it is an act whose quality and efficacy can itself be summarized, diagnosed, and developed through the same means.

Taking this view, the skills involved in executing a skillful evaluation now become a form of meta-assessment; how does one deliver formative assessment of a formative assessment? If considering evaluation as a performance, one can apply the seven principles of evaluation listed above (Nicol and Macfarlane-Dick, 2006) not just to the assessment of performance, but to the assessment of assessment itself. When reframed in this manner, good formative evaluation:

1. helps clarify what good feedback is (goals, purposes, expected outcomes);
2. facilitates the development of self-assessment (reflection) in the feedback given;
3. delivers high quality information to students (i.e. future assessors) about the quality of their assessments;
4. encourages teacher and peer dialogue around providing feedback;
5. encourages positive motivational beliefs and self-esteem;
6. provides opportunities to close the gap between current and desired performance (of feedback delivery);
7. provides information to assessors that can be used to help shape assessment.

With the role of self-regulated learning again at the core of this philosophy, the opportunity to execute the skill to be practiced and improved becomes key. This focus is emphasized in the theory of *experiential learning*, which posits that learning is most effective when students create knowledge through a process of engagement, interaction, and conflict with a rich and holistic experiences (Kolb and Kolb, 2005). If one is to take these two perspectives together—i.e., that evaluation is a skill not only to be learned but also performed—then existing methods of performance training that incorporate experiential learning provide a framework from which new forms of evaluation training and study can be adapted.

The classic form of simulated performance training in music is the dress rehearsal, in which a performance is conducted with every component in place save the audience themselves, thus allowing the performers (and in the case of larger productions, the off-stage support) to ensure that the extra-musical aspects of performance are in place. While this can include testing the practical components of performance—timings, clothing choices, the functionality of electronic or mechanical elements—the performers themselves also have the opportunity to check the technical, physical, and psychological aspects of their craft. Crucially, the dress rehearsal offers the possibility of dealing with the heightened physiological arousal inherent to performance, and its potential to have a maladaptive influence on outcomes should performers interpret this arousal as the manifestation of performance anxiety (Kenny, 2011; Nieuwenhuys and Oudejans, 2012; Endo et al., 2014). This applies not only to the on-stage experience, but also to the period of time spent backstage prior to the performance where performance-related physiological arousal has been found to be at its highest (Williamon et al., 2014; Chanwimalueang et al., 2017). Research has also suggested that the act of video-recording these sessions can also induce anxiety in student performers, again providing an opportunity to simulate the stress of a true performance (Daniel, 2001).

Assessment has been used as a form of experiential learning in educational settings. Indeed, the act of providing self- and peer-assessments as a part of the learning process has seen increased use across higher education, with one meta-analysis demonstrating a trend of strong correlations between peer- and faculty evaluations so long as global criteria are being used (Falchikov and Goldfinch, 2000). In the musical domain, pedagogy classes will investigate theories of teaching and modes of feedback delivery. These may include mock lessons conducted within the classroom or recorded for review by the instructor, which requires sourcing willing students for such experimental teaching. A traditional approach can be also found in the masterclass or studio class, in which the expert musician works with one or more musicians in front of an audience (i.e., the masterclass) or other students (i.e., the studio class; Gaunt, 2017). This basic template can be adjusted to accommodate multiple experts, students taught by their own or other teachers, or, crucially, opportunities for students to critique each other's performance in a controlled setting (Long et al., 2012). While the master/studio class offers obvious benefits for performers (further feedback from a variety of sources, opportunities to perform in public) and for teachers (opportunities to gain exposure as a master teacher, to reach and recruit new students, and to hone their own evaluative skills), those where student feedback is incorporated also provides a platform in which musicians can test and develop their skills of attentive listening and viewing, of performance diagnosis, and of effective feedback delivery (Hanken, 2008, 2010; Taylor, 2010; Long et al., 2012; Haddon, 2014; Gaunt, 2017).

Whether a masterclass or studio class provides specific opportunity to examine the quality of feedback delivery depends largely on the focus and time mandated by the teacher. Otherwise the act of providing an evaluation serves more to enhance reflecting on the performative skill, rather than the evaluative. Studies examining the act of conducting peer- and self-assessments of video-recorded performances highlight performance-focused feedback (e.g., Bergee, 1993, 1997; Johnston, 1993; Robinson, 1993). Daniel (2001) examined video-assisted self-assessment with 35 undergraduate music students at an Australian university, finding in a preliminary questionnaire that fewer than half of the students reviewed audio or video recordings of their own performance with any kind of regularity.

Several studies have examined the act of having students conduct peer-to-peer feedback as part of their training, often examining live pilot programs. Hunter and Russ (1996) worked with an Irish university to develop and monitor a seminar on peer assessment over several years. Students received training in the university's assessment procedures and assembled into panels of students with a variety of instrumental experience, a self-elected leader, and a supporting member of staff who had provided the initial procedural training. In post-evaluation discussions among the students, several extra-performance biases and complications were explicitly raised that have been revealed through subsequent research, including recognition that it was socially and emotionally difficult to provide a low mark despite a weak performance, that assessors playing the same instrument as the performer were harsher in their criticism than those without the specific expertise, that marks assigned often reflected pre-existing expectations of a particular performer (i.e., the so-called halo effect), that the relative relation between the assessor and performer (i.e., whether they were of the same or a different year group) influenced feelings toward providing and receiving the feedback, and panel disagreements were often unresolved due to expedience and a lack of discussion.

Searby and Ewers (1997) examined the use of a peer assessment scheme within courses across a UK university's music programme, starting with an initial pilot in composition and expanding to areas including music performance, business, technology, and theory. In each setting students determined the criteria for assessment, gained initial experience through the evaluation of previous years' work, paired off for peer assessment to be moderated by the lecturer, and received 20% of their final mark for the quality of the written feedback they provided. The process for peer-assessing musical performance was conducted with performances of a different year group rather than previously documented work. With each subsequent year the groups negotiated a new set of evaluative criteria, which follow-on discussion with the students showed to be a critical component of their taking ownership of the evaluative process and thinking critically about creating their own work to be assessed. This feedback on the process also revealed that students were happy with receiving peer feedback and felt that it was a valuable learning tool. Despite hopes that peer-assessment would reduce the evaluative workload of the faculty members, operating the programme did not lead to a significant reduction in their efforts.

Following two studies demonstrating students' inconsistency in their self-and peer-assessment abilities compared with faculty-generated scores (Bergee, 1993, 1997), Bergee and Cecconi-Roberts (2002) assembled experimental groups of three to five undergraduate music majors to perform for one another in four video-recorded sessions, after which they reviewed and discussed the performance footage while completing self- and peer-assessments using fixed rubrics. After self-evaluating recordings of their final jury recitals, these were compared with the evaluations by the jury examiners. No significant difference in ability to self-evaluate was shown based on year or performance level, and correlations between self- and faculty assessments were modestly higher among the experimental group compared with a control group who had not completed the peer assessment discussion sessions. However, a great deal of variability remained in the scores, especially in ratings of tone and interpretation. A follow-up experiment that included greater discussion of the evaluative criteria and their application to two sample scores also showed moderate to no effect of the treatment on alignment of self- and peer-assessments with faculty assessments, with the authors suggesting that the interventions had not fully engaged with the social and environmental complexities of performance self-assessment.

Daniel (2004) had 36 students who were involved in weekly performance seminars provide feedback on fellow student performances in the form of short evaluative comments and as detailed grades using a segmented scheme. Reflective questionnaires showed that students preferred the structured approach and that those too reserved in their critical judgments tended to improve over the course of the sessions.

In Blom and Poole's (2004) research, 16 third-year music students were asked to evaluate second-year performances in an Australian university. Having completed self-assessment tasks in their first year and paired peer-assessment critiques in their second, they were tasked with grading recorded performances of their second-year peers using the same criteria employed by staff, providing written critiques to be read by the performers, assigning grades, and providing a self-reflective commentary on the process. Students struggled to cope with the variety of instrumental specialties they were asked to assess, the prospect of delivering harsh feedback when they already had a personal familiarity with the performer, adhering to a pre-existing set of criteria, and their ability or “authority” to provide such assessments to their peers. As Hunter and Russ (1996) demonstrated, the students found the exercise to be helpful in not only developing their abilities and confidence in assessment but also how they might adjust their performance for assessment. Further research also followed on Hunter and Ross' use of student-chosen evaluation criteria, finding that students placed focus on “soft” skills in assessing rehearsal quality—personal, interpersonal, and organizational skills—and “hard” skills in assessing performance quality: technical, analytical, and musicianship skills (Blom and Encarnacao, 2012).

Lebler (2007) described the establishment of a “master-less studio” in the execution of a course on popular music production at an Australian university in which students self-directed their learning strategies, outcomes, and outputs in collaboration with their peers. This included a structured method of peer evaluation in which recordings were shared and written commentary posted on a course website, amounting to over 180,000 words of feedback on 292 recorded tracks in one semester. Course conveners monitored whether the feedback conformed to good standards of constructive criticism, highlighting instances of overly authoritative tone or lack of appropriate detail, although specific instruction or focus on effective feedback production was not provided.

Latukefu (2010) examined a scaffolded peer-assessment framework among undergraduate vocal students at an Australian university. Adapting the model set by Searby and Ewers (1997), student focus groups established the assessment criteria and processes before the programme was implemented across a cohort. Following dissemination and discussion of the criteria to a class on contemporary performance practice, panels of three students performed peer evaluations. An open-ended survey found that students recognized the benefits of peer evaluation in improving their abilities to reflect upon their own performances, as well as developing skills important to their future work as evaluators. They highlighted the difficulties in conducting these evaluations with peers and friends, citing awkwardness and social influences preventing objective discussions of performance and assessment.

The Center for Excellence in Music Performance Education at the Norwegian Academy of Music established peer learning and group teaching as a “principal instrument study” (Hanken, 2016). Several approaches were employed, each a variation on a teacher-supervised studio class in which students engaged in discussion of performance and feedback. One approach employed Lerman and Borstel's (2003) Critical Response Process, which comprises an initial discussion of what components of the performance are meaningful, the performer asking questions on which they would like feedback, the evaluators asking neutral questions of the performer, and finally the evaluators asking permission to give opinions on specific aspects of the performance, only delivering those opinions if asked. This study found that, in the most effective uses of the method, the fourth stage became redundant as the performer had already reached the relevant conclusions through the dialogue. Hanken also highlighted the role that peer learning can play in continuing professional development of music teachers through seminars and discussion, combatting the isolation that can be inherent to music instruction through the nature of working practices.

More recently, Mitchell and Benedict (2017) employed peer-to-peer examination as a teaching tool during auditions at an Australian university. Rather than having the students provide evaluations in genuine grading scenarios, they rated live performances with or without a blinding screen in front of the stage, as well as recorded performances in audio only, visual only, and audio-visual scenarios to confront directly the issues of audio/video interaction inherent to music performance evaluation. The student judges felt more confident when rating performances in audio-only conditions and were prompted to reflect on the role of their appearance and stage presence in their own performances.

Finally, Dotger et al. (2018) adopted methods used in medical education to train physicians, targeting a specific form of feedback delivery in music teachers. Where a doctor may interact with a mock patient, the researchers had 13 trainee music teachers interact with a mock parent, herself coached to question the teachers as to why her daughter had not been successful in a recent (hypothetical) audition, the validity of the assessment itself, and whether her daughter had “the look” (i.e., whether she conformed to the presumed stereotypes of performer appearance). Trainees had not been given prior instruction in how to navigate the interaction, thus their responses were highly variable. Several were able effectively to incorporate a combination of personal experience, acknowledgment of the parents' concerns, and specific advice for further development into their conversations.

In reviewing these approaches, several similarities can be seen. Each embraced experiential learning, not only giving students the ability to take part in the act of evaluation but in several cases also taking control over the terms and goals of the process. Those that captured outcomes found positive responses from the students and educators. However, simply providing learners the opportunity to evaluate others is not so simple a proposition, with several of the studies highlighting the workload costs of administering such training and acknowledging that many still felt unprepared to face the pressures of genuine evaluation situations. It is here that the gap is highlighted between artificially constructed assessments among familiar peers and settings and the heightened competitions, auditions, exams, and masterclasses in which the students will be called upon to make impactful decisions. Alternatively, allowing learners (or researchers) access to true evaluative situations robs them of control of the situation and risks affecting the outcomes of those to be evaluated, especially if the evaluators in question are novices.

What is needed, therefore, is a way to recreate the complexity of a true or mock evaluation while maintaining control over the stimulus and setting to be evaluated. In the mock-parent study by Dotger et al. (2018), the authors describe the approach as a form of simulation, differentiating it from a role-playing exercise in that those taking part were told that the mock parent would never break from their character, and that the interaction could not be stopped or tried over. An existing approach embracing the concept of simulation can be found in the use of Immersive Virtual Environments (IVEs).

### Simulating Performance

#### Immersive Virtual Environments (IVEs) and Distributed Simulation

IVEs comprising some combination of projected visuals, aural and acoustic simulation, interactive physical environments, and closed narrative loops have now seen decades of use in both medical and social psychological settings (Blascovich et al., 2002a; Sanchez-Vives and Slater, 2005). The simulation of performance as a training tool has seen considerable use in non-musical domains, including the development of pilots (Hamman, 2004), athletes (Miles et al., 2012), and firefighters (Bliss et al., 1997). A particularly fruitful domain has been that of medicine, where shrinking opportunities to gain experience with patients in consultation and surgery, the unending and exponential growth of clinical techniques to be learned, and increased pressure to reduce the amount of practicing skills on patients is driving a shift to learning through simulation (Kneebone et al., 2010). While their efficacy was initially contested (Blascovich et al., 2002b), simulations can offer insights into issues of human perception and social behavior, and their functionality has increased with the rapid growth in computational power and projection techniques. Furthermore, their ability to simulate risk while providing the operator with complete control over the environment has demonstrated their efficacy as a therapeutic tool to combat, for example, posttraumatic stress (Difede et al., 2002), and fear of flying (Rothbaum et al., 2000), spiders (Bouchard et al., 2006), and public speaking (Slater et al., 1999).

One branch of this work has been the advancement of distributed simulation, wherein alternatives to the advanced, complex, expensive, and/or immobile architectures that often typify simulation environments are developed that emphasize affordability, accessibility, and portability (Kneebone et al., 2010). In Kneebone et al.'s example, a surgical theater is reproduced in an affordable, inflatable room; expensive equipment is represented through life-size, high-fidelity photographs; lightweight versions of surgical lighting provide the intensity of a lit operating table; speakers recreate the genuine sounds of the operation space; a combination of affordable prosthetics and human actors provide the social, visual, and tactile experience of engaging with a patient. This approach emphasizes recreating the function, rather than the structure, of the true environment, with particular focus on the aural and visual stimuli peripheral to the central task and has been found to be an effective and adaptive form of training (Kassab et al., 2011). The affordable and portable nature of this approach, in particular, lends itself to the musical domain, where space and funds are regularly in short supply in music education institutions.

#### Simulating Music Performance

Several approaches to simulated performance training through IVEs have been employed in music research. Orman (2003, 2004) used a head-mounted display in which she simulated an empty practice space and seated audience of familiar peers, faculty members, or the head of bands performing an audition. Tests with eight saxophonists showed some evidence of increased heart rate in several participants, although results were inconclusive due to lack of correspondence with physiological scales and lack of experimental control. Bissonnette et al. (2011, 2015) had nine guitarists and pianists perform six sessions in a virtual environment comprising a classical music audience and/or panel of three judges giving a variety of reactions and interjections presented via four large screens in a three-dimensional arrangement, speakers, and stage lights. When state anxiety scores were taken following public performances before and after these sessions, participants with high trait and initial state anxiety showed a reduction in state anxiety across the two performances significantly greater than those of a control group who had not experienced the virtual environment. Significant increases in third-party-assessed performance quality were also noted in the experimental group. Further study tracked changes in reported anxiety within each of the six 1-h sessions, finding a decrease in anxiety provoked by the simulation in subsequent sessions so long as similar musical material was being presented (Bissonnette et al., 2016).

A different immersive approach to the simulation of musical performance can be seen in the development and operation of Williamon et al.'s (2014) *Performance Simulator*. The platform recreates an intimate concert recital with 24 audience members or an audition for a panel of three expert judges. To create the audience, 11 participants were filmed via green-screen performing typical random movements of concert viewing, as well as providing specific responses (e.g., mild applause, booing, a standing ovation, etc.). Accompanying audio was recorded separately. This footage was then compiled into a digitally constructed representation of a concert space, which was itself embedded into a software programme that allows the operator to trigger the various reactions, in addition to cuing coughs and mobile phone rings intended to test the performer's concentration. For the audition simulation, three professional actors were recorded while seated at a table recreating the effect of an audition panel. Following a neutral greeting to the performer, they can be activated to provide an overtly positive, neutral, or negative mode in their passive listening, conveyed through eye contact, facial expression, and body language, and in a triggered final response.

Following Kneebone et al.'s (2010) goals of distributed simulation, the goal of the *Performance Simulator* was to replicate not only the panel or audience, but also the surrounding environment. In addition to the stage lights as used in previous simulations (Bissonnette et al., 2011, 2015), curtains were placed alongside the screen and a darkened, stage-light atmosphere replicated in the room. A backstage area was also recreated including dimmed lighting, music stands, seating, audio bleed from the stage comprising indecipherable chatter for the audition panel and the sound of an audience taking their seats for the concert setting, the latter of which was also featured backstage on CCTV footage of a comparable performance space and audience. An operator played the role of a “backstage assistant,” guiding the performer through the experience while operating the virtual panel or audience. Crucially, this actor interacted with the performer as though the event were a genuine performance, and the performers themselves were expected to come wearing concert dress and to allow themselves to be caught up in the experience. Examination of electrocardiographic and self-reported state anxiety data among seven violinists demonstrated that the simulation provoked stress and anxiety responses comparable to a live audition, and further qualitative research found that students perceived the simulation to be an effective tool to provoke and train for performance anxiety (Aufegger et al., 2017).

This work was followed by Glowinski et al. (2015) in which the projected audience comprised fully-digitized audience avatars standing in loose formation in a large, simulated concert space and projected in an immersive, three-dimensional configuration. As the audience members were rendered in real time it allowed the operators to manipulate the audience's behavior; in this case, the audience's “engagement” was manipulated via altering the proportion of avatars fixing their eye gaze on the performer versus those whose gaze moved randomly and disinterestedly through the space. Using this, the researchers were able to demonstrate through motion tracking how four violinists' performance movements were altered, although not consistently, under different audience conditions.

Based upon these existing simulation approaches, this article presents the novel conceptualization and development of a prototype tool to apply the concepts of Virtual Immersive Environments and distributed simulation to the practice and study of music performance evaluation.

## The Evaluation Simulator

There is a clear need for further approaches to study the act of live performance evaluation in a controlled environment and to improve and expand the delivery of assessment training. Musicians require access to skilled evaluators to provide feedback on their own performance and to develop skills as assessors to prepare for portfolio careers and enhance their self-evaluative abilities. Teachers and educational institutions have a duty to ensure they are preparing their students for careers that include teaching and assessing and to ensure that the evaluations they provide of their students are fair and robust. And researchers require new means to investigate and control experimentally the myriad social and environmental factors that influence the act of decision-making.

While numerous approaches have been described that apply the tenets of experiential learning and simulation through mock experience, none have embraced the possibilities of IVEs or distributed simulation in recreating the surrounding and intensifying stimuli of the true evaluative experience. This is akin to the pianist experiencing a “performance” in a closed room with their peers, minus the time backstage, the concert dress, the darkened hall, the stage lights, the unfamiliar audience, and the true pressure of a live performance. It is these features that music performance simulations have sought to replicate. A genuine performance evaluation, as discussed above, can come with the same pressure of performance. Increased arousal can limit the ability to attend to and process information (Hanoch and Vitouch, 2004), which is also central to the act of performance assessment. Thus, the goal of the present work was to develop an immersive simulation that stimulated the heightened pressure of performing an evaluation, to allow for immersive and experiential training while providing a controlled setting to facilitate experimental research.

To address these goals, the *Evaluation Simulator* was developed as a prototype to allow for the recreation of the following scenarios in training and research:

1. evaluating an expandable set of replicable stimuli;
2. evaluating alone or as part of a panel;
3. evaluating in a heightened setting, such as in a live competition or masterclass, where the judges themselves are a focus of attention;
4. having to evaluate a performance of good or poor quality;
5. having to deliver summative, diagnostic, and/or formative evaluation directly to the performer immediately and verbally;
6. having to deliver that feedback to a performer who is in a variety of emotional states.

## Development

A primary question in developing the simulation was in the fundamental mode of stimulus presentation—that is, how the performance would be immersively visualized. The music performance simulation literature presented three existing approaches: (1) a head-mounted virtual display (Orman, 2003, 2004), (2) a projected visualization of 3D rendered avatars (Bissonnette et al., 2011, 2015), or (3) a projected visualization of looped video recordings (Williamon et al., 2014). The head-mounted display, while offering perhaps the most “immersive” of the approaches, was discounted due to the difficulties in engaging multiple people simultaneously with the simulation and the relative complexity and cost in developing and operating the platform. A system employing a large display or screen and projector typical to education settings was thus determined to be the most appropriate for the intended use cases.

With regard to artificially-rendered avatars, they provide several advantages: (1) they allow for complete control over audience behavior, reactions, and appearance, theoretically providing infinite variety in audience conditions; (2) they provide the opportunity to generate audiences that are dynamically reactive to the performer, altering their behavior as a true audience might in response to the quality and expressiveness of the performer [a stated objective of Glowinski et al's (2015) research]; and (3) they theoretically allow for seamless transitions between presentation modes (e.g., a stationery to an applauding audience) as transitions can be rendered in real time, where use of video often necessitates noticeable transitions or “jumps” between sets of pre-recorded footage. However, such an approach comes with drawbacks. Despite exponential advances in the ability to create lifelike human avatars and repeated demonstration that they can provoke realistic responses, they tend to remain across the “uncanny valley” that separates them from being perceived as true human representations (de Borst and de Gelder, 2015; Kätsyri et al., 2015). This has particular salience in music performance evaluation considering the highly influential role of the performer's behavior and appearance in performance evaluation (Platz and Kopiez, 2012). The use of pre-recorded video loops eliminates this problem and allows for photorealistic performers. With a carefully controlled protocol and instructions, it offers the possibility of convincing users that they are interacting with a genuine audience or auditioner via a videoconferencing system.

Considering the limitations of these technologies and of existing practice described throughout this article, 10 qualities were determined as crucial in development of the *Evaluation Simulator*. These were as follows (and are summarized in Table 1):

1. **Experimentally replicable:** Replicability was the primary goal of the simulator, i.e., providing experiences that could be duplicated within and across students or study participants. This would not be possible in mock or true performances, and while assessing lone recordings allows for replicability of the evaluative experience, an IVE is necessary to immerse the judge in a stimulating environment.
2. **Immersive:** The experience must be free from extraneous distraction and provide a full sensory experience of the evaluation. Mock evaluations offer potential here, if a suitable environment is created, although IVEs specifically tailor this experience.
3. **Heightened arousal:** The immersion should seek to increase the arousal experienced in completing the evaluation, mirroring the risk of the true situation. Again, mock evaluations have the potential to recreate this, although examples in the literature are lacking.
4. **Risk-free for performer/organization:** Conducting genuine evaluations defined by real impact on the grades/standing of the performer introduces risk for those being evaluated. A simulation should recreate this tension while avoiding the need to influence actual assessment procedures.
5. **Photorealistic:** Due to the importance of visual performance features, looped recorded video within an IVE would be ideal as used in Williamon et al.'s (2014) *Performance Simulator*.
6. **Allows solo and group evaluation:** The simulator should allow a panel of evaluators to interact in a genuine physical environment. This is a particular challenge for VR applications, which naturally isolate the user within the head-mounted display.
7. **Inexpensive to create:** To determine an approach that could be widely adapted following the goals of distributed simulation, the complex computing expertise and equipment required to generate immersive VR or computer-generated avatars precluded their use in this simulator.
8. **Inexpensive to operate:** The equipment required for the employment of VR simulation is not readily available in most music learning environments. Mock evaluations have the potential to incur great expense if performers/actors need to be hired.
9. **Adaptable:** True performances are restricted by nature. Mock evaluations and simulations rendered in real time offer infinite adaptability. While video simulations are more restrictive in their adaptability, multiple scenarios could be filmed in advance and combined to allow an exponential number of possible use cases in combination with variations in the environment.
10. **Portable:** The experience must be operable in a wide variety of physical locations, with minimal effort and cost required in transporting it.

**Table 1:** The qualities of traditional and immersive virtual environments (IVEs) in the training of evaluative skills and in research.

**Needs of the evaluation simulator**	**Traditional environments**			**Immersive virtual environments**		
	**Video review**	**Mock**	**True**	**VR display**	**3d display**	**Looped video**
Replicable	Yes	No	No	Yes	Yes	Yes
Immersive	No	Potential	Yes	Yes	Yes	Yes
Heightened arousal	No	Potential	Yes	Yes	Yes	Yes
Risk-free for performer	Yes	Yes	No	Yes	Yes	Yes
Photorealistic	Yes	Yes	Yes	No	No	Yes
Solo and group eval.	Yes	Yes	Yes	No	Yes	Yes
Inexpensive to create	Yes	Yes	Yes	No	No	Yes
Inexpensive to operate	Yes	Potential	Yes	No	Yes	Yes
Adaptable	Yes	Yes	No	Yes	Yes	Yes
Portable	Yes	Yes	No	Yes	Yes	Yes

Table 1 summarizes these points and the degree to which traditional evaluative environments used in research and teaching (assessing recorded videos, mock evaluations, and true evaluations) and the options for IVEs described earlier (VR displays, 3D rendered displays, and looped video displays) meet the demands. As a result of this summary, it was determined that Williamon et al.'s (2014) *Performance Simulator* provided the best model upon which to base the *Evaluation Simulator*. To achieve this, performance footage would need to be recorded, combined in an interactive software framework, and presented within an artificially created physical and social environment. This process is outlined below.

### Recorded Video

#### Stage and Setup

The stage setting was designed to be ambiguous in the size of the space in which the performer was appearing, allowing the simulation to be physically displayed in a variety of settings without creating visual conflict. To achieve this, the video was shot against a black-curtained backdrop without side walls or ceiling visible, leaving the size of the space ambiguous. A carpeted floor was also chosen to maximize transferability to alternate spaces, as this could be interpreted as a rug placed over the local flooring. A long shot was used, maximizing the size of the performer in the shot while ensuring his entire body remained in frame at all times. This served several purposes: (1) guaranteeing the whole body could be seen without cut-off to give the strongest impression of a performer in the room with the evaluator; (2) allowing the assessor to judge the full range of body movement; (3) maximizing the size of the instrument and hands to facilitate instrument-specific technical assessment; (4) maximizing the size of the performer's face to facilitate social cues; (5) allowing the performer to be projected as close to life-size as possible on a standard, stand-mounted projector screen to facilitate the simulation; and (6) minimizing the perceived distance from the performer to allow for a more socially intense setting.

**Figure 1:**
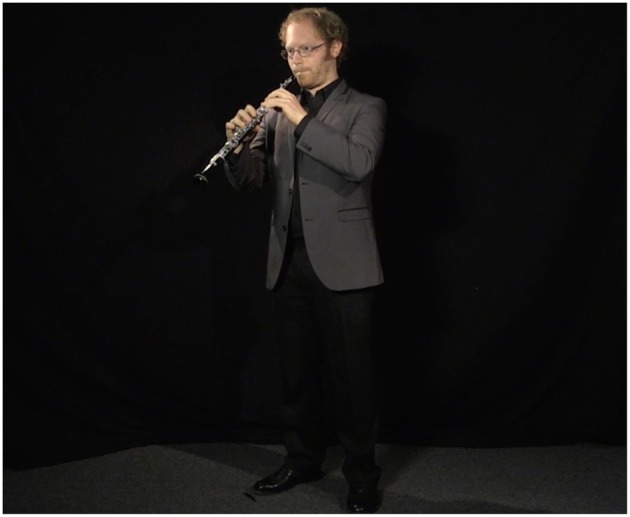
Framing of the performer in the recorded video. The size of the performer in the scene was maximized to enhance the effect of the simulation.

Professional studio lighting and audio/video capture equipment (with a close-mic stereo setup) was used to maximize the veracity of the videos and facilitate the simulation. The performer was asked to wear semi-formal clothing appropriate for a high-level orchestral audition (see Figure 1).

#### Performance Footage

**Figure 2:**
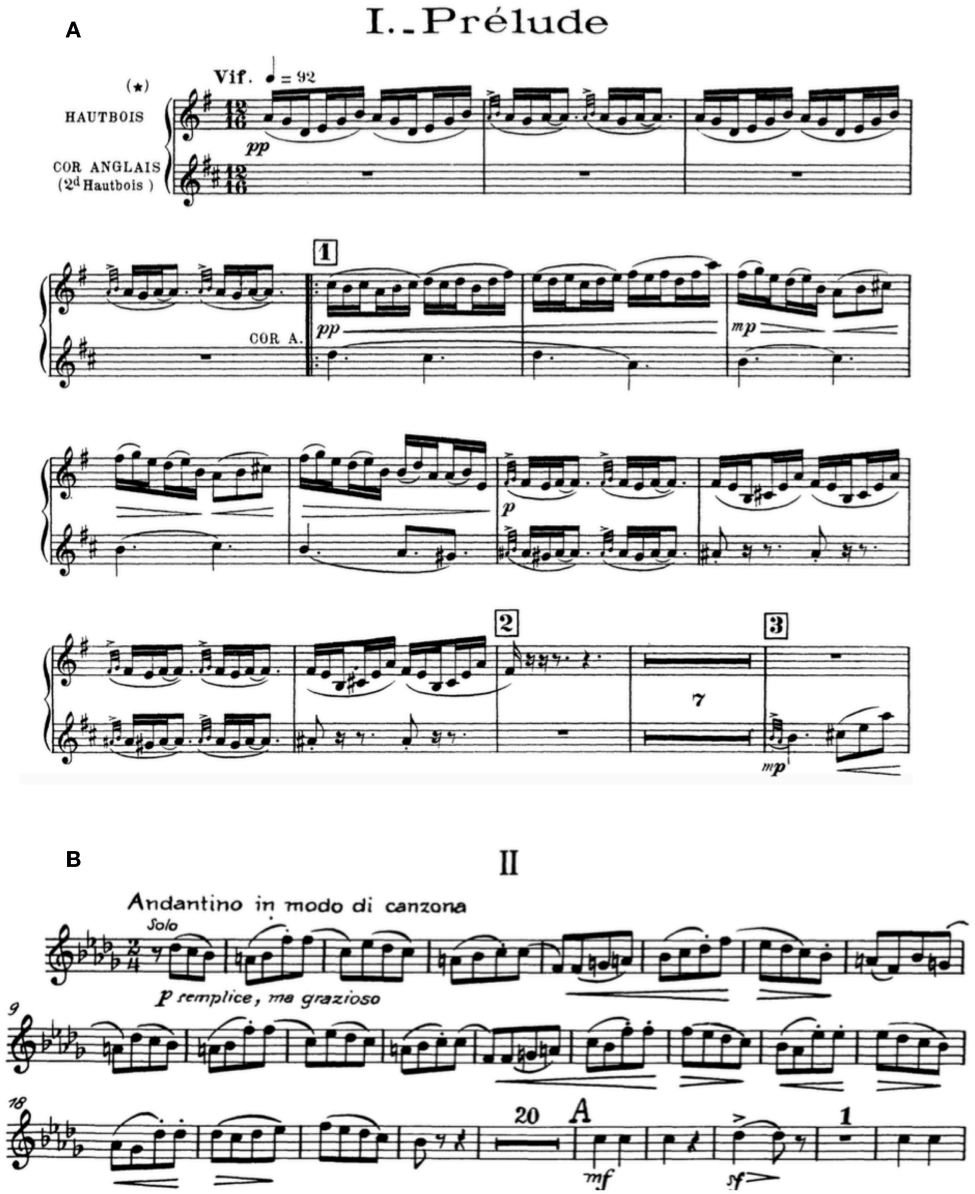
Musical excerpts recorded for the simulation. Top panel **(A)** oboe solo from the *Prélude* of Maurice Ravel's *Tombeau de Couperin*, bars 1–14 (Ravel, 1919, p. 1); bottom panel **(B)** oboe solo from the second movement of Tchaikovsky's *Symphony No. 4, Op. 36*, bars 1–21 (Tchaikovsky, 1946, p. 6).

The performer, a semi-professional oboist, was asked to prepare two excerpts of standard orchestral repertoire typical of a professional audition. The excerpts were chosen to vary in tempo and style: a relatively fast work emphasizing articulation, ornamentation, and rhythmic drive, and a relatively slow work to demonstrate melodic phrasing and breath control. Respectively, these were the oboe solo opening of the *Prélude* of Maurice Ravel's *Tombeau de Couperin*, bars 1–14, and the oboe solo opening of the second movement of Tchaikovsky's *Symphony No. 4, Op. 36*, bars 1–21 (see Figure 2). For each work the performer delivered two performances for a total of four: a “good” performance of high playing standard, and a “poor” performance in which he struggled with intonation, tempo, and tone and displayed mild facial frustration.

#### Extra-Performance Footage: Entrance, Feedback, and Exit

The beginning of each of the four recorded performances opened with the empty stage, followed by the performer walking in and standing on a mark facing the camera. In each case, the performer was asked to face the hypothetical judging panel, wait ~3 s to leave time for a brief welcome and indication to start, give a nod of acknowledgment, then begin performing. The same activity was recorded ahead of each of the four performances.

**Figure 3:**
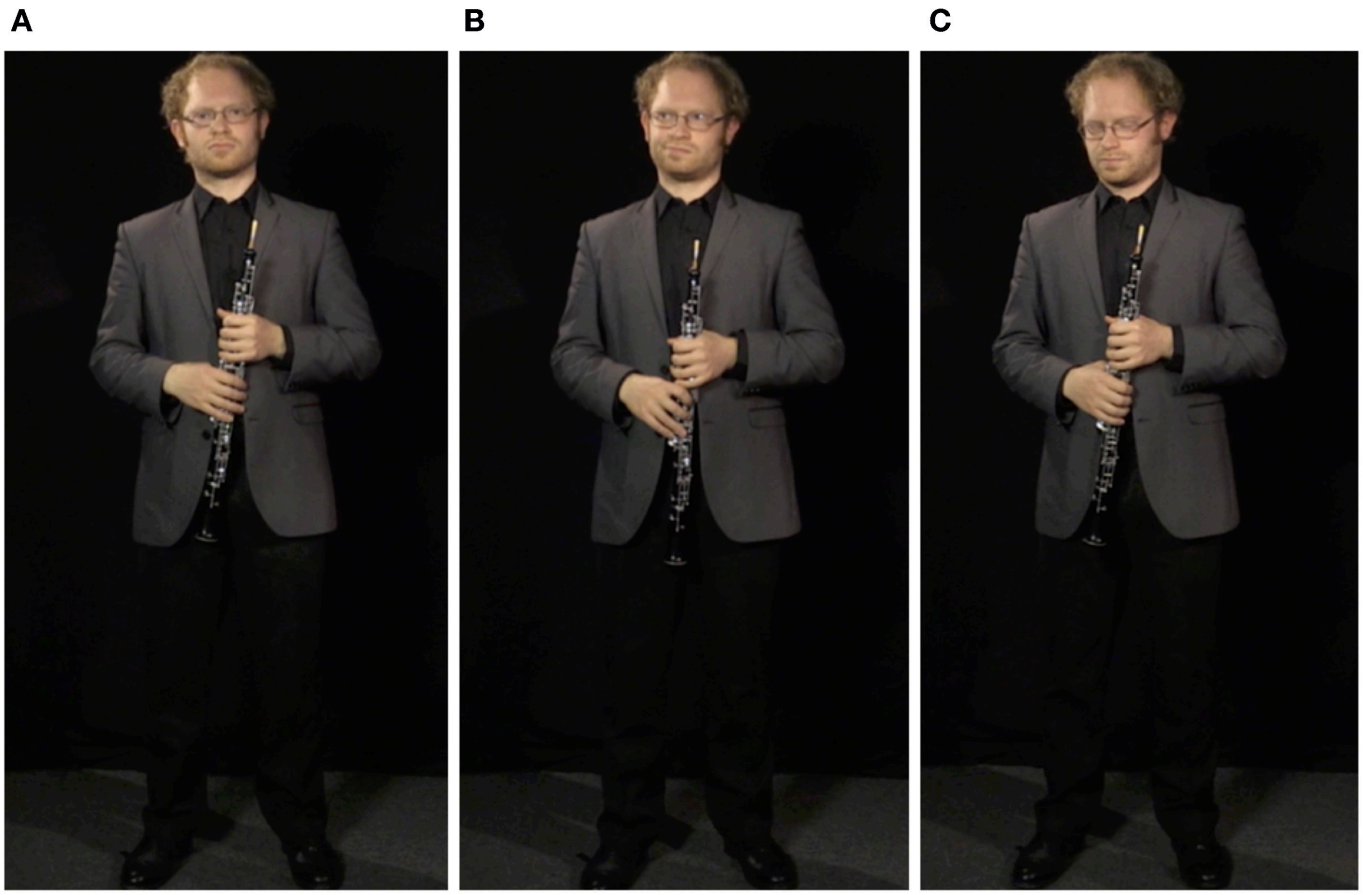
Screenshots of the performer's three reaction modes. **(A)** *confident*. **(B)** *frustrated*. **(C)** *distraught*. These reactions can also be seen in Supplementary Videos 1–4.

Following the performance, the oboist was asked to face back toward the panel to receive feedback. At this point, three modes of feedback reception were filmed, chosen by the authors to represent a variety (though not an exhaustive list) of potential positive and negative performer reactions: (1) *confident*, in which the oboist was instructed to appear resolute and stoic, ready to receive positive or negative feedback in stride with direct eye contact and occasional nods of understanding; (2) *frustrated*, in which he was asked to appear disappointed in his performance and to not give the panel his full attention, avoiding eye contact and punctuating his reaction with subtle eye rolls, sighs, and grimaces; and (3) *distraught*, in which he was told to appear in a poor emotional state following the performance, looking at the floor and giving the impression of holding back tears with the expectation that poor or harsh feedback would be given (see Figure 3). Each feedback scenario was recorded for 60 s, with the performer instructed not to change standing position and minimize torso movement to allow the segment to be looped (described further below). Each of the three feedback scenes was concluded by the performer saying “thank-you very much” or “thanks” to the panel in the style of each setting—confident and gracious, brief and dismissive, barely audible and distraught—and walking out of frame in the direction he entered.

A summary of the seven pieces of video footage collected can be found in Table 2, and examples of the video files themselves can be downloaded as Supplementary Videos 1–4,^2^, which show the performances and uncut performer reactions that would be looped (or cut short) in the final simulation experience, depending on the simulated scenario. Screenshots of the three performance reactions (*confident, frustrated*, and *distraught*) are shown in Figure 3.

**Table 2:** Summary of the video footage collected.

**Video**	**SV Code**	**Category**	**Description**
Perf 1A	Video 1	Entrance and performance	Ravel (fast), good quality
Perf 1B	Video 2	Entrance and performance	Ravel (fast), poor quality
Perf 2A	Video 4	Entrance and performance	Tchaikovsky (slow), good quality
Perf 2B	Video 3	Entrance and performance	Tchaikovsky (slow), poor quality
React A	Video 1	Reaction and exit	*Confident*
React B	Video 2	Reaction and exit	*Frustrated*
React C	Video 3	Reaction and exit	*Distraught*

*In the Evaluation Simulator, any “Entrance and performance” may be paired with any “Reaction and exit,” allowing for 12 possible permutations. Samples of these videos are available to download as Supplementary Files (as compressed files, with decreased audio/video quality), where the three reactions and stage exits are paired with three of the performances in Supplementary Videos 1–4 following the codes (SV) in Table 2*.

### Software

**Figure 4:**
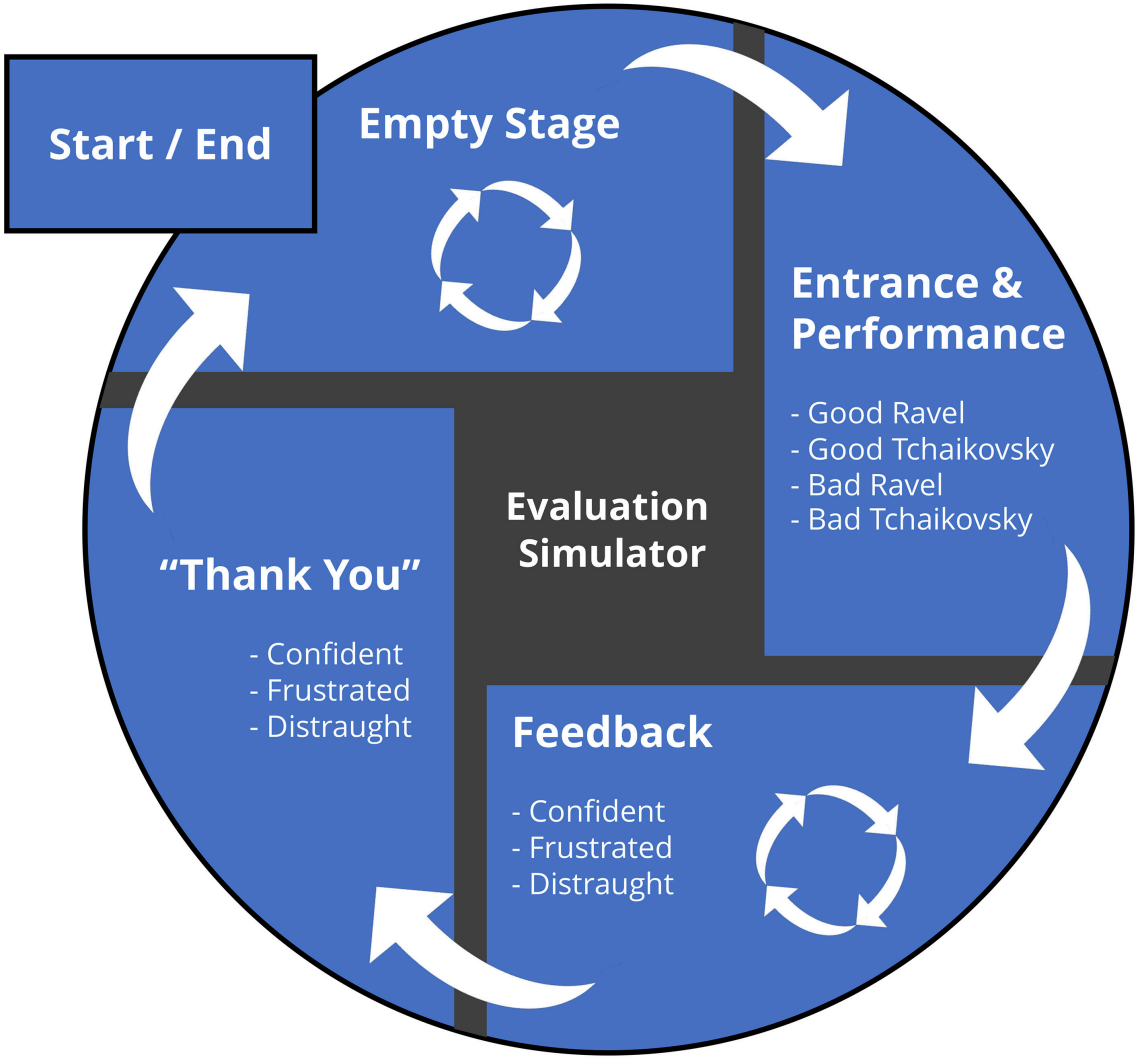
Process mapping of the software interface. Following a hold of the empty stage shot, pressing keys 1–4 triggers the stage entrance and respective performance. During this performance, selecting the “b” or “c” keys prompts the eventual transition to the appropriate reaction (which otherwise goes to the default *confident*). Once the looped feedback reaction is no longer needed (which can be quickly skipped in scenarios in which no immediate verbal feedback is provided), the space bar triggers the stage exit and returns the software to the original stage, ready for another evaluation.

Figure 4 outlines the interaction mapping of an Adobe Flash-based software interface developed to manipulate the videos using keyboard commands. Upon opening the program (and setting to full-screen view), the software holds a still image of the empty stage. By pressing keys 1–4 the operator triggers one of the four recorded performances (i.e., Ravel vs. Tchaikovsky; good vs. bad), which triggers the stage entrance and performance. Following the performance, the neutral reaction is then triggered by default with a dissolve transition between the two consecutive videos; the operator can trigger the *frustrated* or *distraught* reactions by pressing the “B” or “C” keys at any point following the beginning of the performance. The last key pressed triggers the corresponding reaction, and the “A” key returns the reaction to *confident*. Once one of the reaction videos have been triggered, it remains on a continuous loop until the operator closes the session by pressing the space bar, which triggers the corresponding “thank you” and the performer's exit sequence.

The interface can also be operated using a standard USB presentation remote. In this case, the equivalent of a slide advance triggers the “good” Ravel performance with a confident reaction, and another click triggers the stage exit. This can also be used to end any of the reaction loops if they had been triggered by the computer keyboard.

### Physical Environment

**Figure 5:**
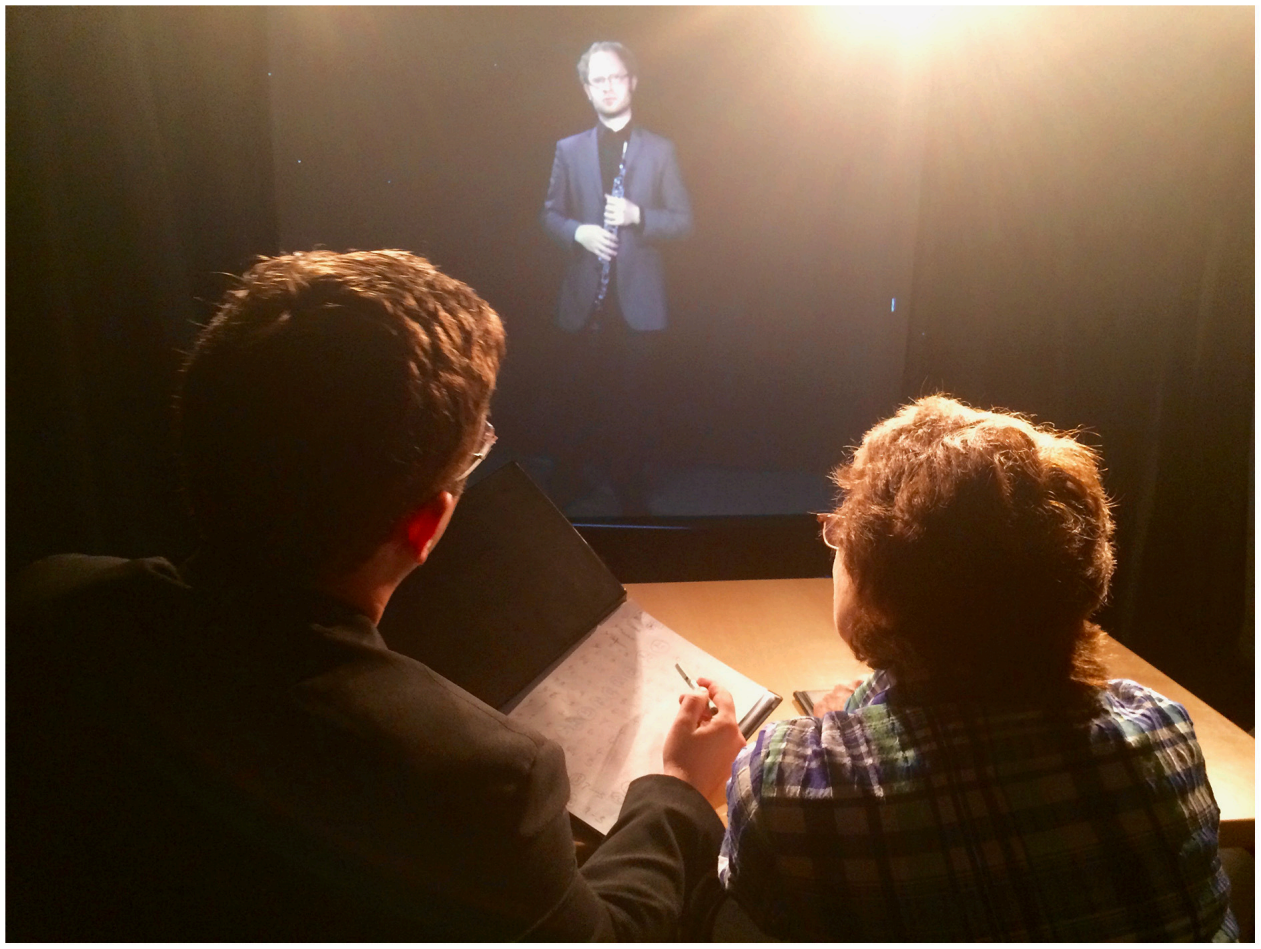
Two evaluators delivering performance feedback in the *Evaluation Simulator*. Stage lights illuminate a user and a facilitator in the environment, delivering feedback to the performer in the *confident* feedback mode.

While the recorded video and software interface provides the core simulator experience, it is augmented by features of the physical environment in which it was designed and into which it can be set up. The configuration used here mirrors that of Williamon et al.'s (2014) *Performance Simulator*. The projection screen (or large monitor) is placed against a wall and flanked by heavy curtains, giving the impression of a stage space extending beyond the physical room. Where possible, the screen is large enough to display the performer at a 1:1 scale and placed at floor level to give the impression of the performer standing in the room; where the screen must be raised, the gap at the bottom can be blocked to give the impression that the performer is standing on a raised platform or stage. The curtains and screen are topped by remote-operated stage lights, directed back at the panel to heighten the feeling of attention and pressure on the decision-making process. The room is best left darkened to maximize the effect of both lights and projection. High-quality speakers are placed as close to the projection as possible to give the impression of the performance emanating directly from the virtual performer. A table and chairs for the panelists are placed at the center of the room, to which props can be added that are common to a judging experience (e.g., glasses of water, clipboards, judging rubrics, desk lighting; see Figure 5).

### Operation

A crucial component of the simulation is the human operator and the supporting theater he or she provides; the operator must treat the situation as a genuine performance and not allude to the artificial nature of the environment, emphasizing the role of simulation over role-play (Dotger et al., 2018). The details of the operator's role can alter based on the specific setting, but generally comprises a welcome and introduction, briefing on evaluation protocols, orally calling in the mock performer (with accompanying triggering of the stage entrance sequence and desired performance sequence), triggering the desired reaction sequenced if not the default, triggering the stage exit at the appropriate point (this may be immediately following the performance and before any performer reaction should the simulation situation not include verbal feedback from the panel), and providing the closing and debriefing of the user. The operator may be serving alongside a researcher, teacher, and/or one or more mock panelists performing their respective roles, or they may be serving these roles themselves.

### Initial Piloting

The simulator was piloted at the 2015 Cheltenham Music Festival, where it was set up as part of a public engagement event to allow festivalgoers to experience the heightened effect of performing as a competition judge akin to those popularized by the *Idol, X Factor*, and …*'s Got Talent* series. This also provided an opportunity to test the simulator's functionality as a piece of distributed simulation in whether it could be set up quickly in a space not designed for such use and provide an effective simulation. The collapsible lights, curtains, and projection screen and portable projector were assembled in a darkened storage room, with table and chairs locally sourced. Three operators facilitated the event: one to greet, brief, and debrief guests on their experience, one to act as a fellow panelist to the guest and prompt them to provide feedback to the performer, and one to operate the simulation from backstage. Public response was positive, with guests highlighting the intensity of the experience and several questioning whether the performer in question had been video conferenced in due to his coincidental “reactions” to statements they had made in their feedback. While further validation is required, this pilot suggested the goals of immersion, increased arousal, adaptability, portability, and cost-effectiveness to operate was achieved.

## Applications and Discussion

The benefits of IVEs and distributed simulation have already been seen in the domains of medical and music performance training, providing new avenues to promote experiential learning and provide a platform to conduct performance research in controlled environments. The *Evaluation Simulator* provides the first opportunity to apply these benefits to the study and training of music performance evaluation. As the adaptability of the software and surrounding social environment provides a variety of permutations, potential applications can be posited for its use in teaching and research.

Before addressing these possibilities, it is important to highlight a central limitation of the simulator at this early stage of development. While it was created with the goal of stimulating heightened arousal, a full efficacy study will be required to demonstrate whether the simulator is truly capable of evoking similar evaluative and physiological responses to genuine evaluation settings, as was demonstrated with the *Performance Simulator* (Williamon et al., 2014). Such work, however, would be complicated by a lack of knowledge of the real-world analog. While much is known about musicians' responses to performance situations (e.g., Kenny, 2011; Nieuwenhuys and Oudejans, 2012; Endo et al., 2014; Williamon et al., 2014; Chanwimalueang et al., 2017), no work to date has examined the physiological experience of the music examiner or competition judge. A major line of research is required to achieve this aim, one in which the *Evaluation Simulator* could play a central role. A second limitation is the range of performances available for evaluation: while quality and response can be varied across the two performances for a total of 12 evaluation scenarios from the videos alone, they are nevertheless restricted to one performer on one instrument with two pieces of standard repertoire. However, the existing conceptual and software framework could be expanded with relative ease, requiring only the collection of new video footage with different performers (including variation in extra-musical features such as appearance, dress, and behavior), instruments, and repertoire while following the same script of entrance, performance, feedback, and exit footage. The descriptions above provide guideline principles for how this footage can be collected, with an emphasis on maximizing audio/video quality and veracity of the performance situation. Over time a library of performances could be assembled, and even shared between groups or institutions following a similar framework.

### In Pedagogy

Care must be given in how best to employ the simulator in pedagogical settings. Through a review of studies in the medical domain, Issenberg et al. (2005) outlined 10 good practices in using simulation in training settings. They highlighted how (1) feedback should be given during the learning experience, (2) learners should practice their skills repetitively, (3) simulators should be integrated into the overall curriculum rather than used in extra-ordinary circumstances, (4) learners should practice with increasing levels of difficulty, (5) simulators should be used with a variety of learning strategies, (6) simulators should capture a variety of contexts, (7) learning should occur in a controlled environment, (8) learners should be provided with individualized experiences, (9) clear outcomes and benchmarks should be provided, and (10) the validity of simulators should be demonstrated. In its current form the *Evaluation Simulator* fosters repetition (2), a range of difficulty (4; i.e., the differing performance qualities and responses) and the controlled environment (7). The need to validate the simulator (10) has already been discussed, as has the possibility to expand the simulation to a wider variety of contexts beyond what is already possible through variations in the software interface, social, and environmental factors (6). The use of varying strategies (5) while providing individualized learning (8) will be up to the instructor, who can vary the use of group size or use of instructor-vs-peer led settings. For example, a lesson might have students enter alone, with the instructor as a panel leader, with a panel of peers, or with a panel of strangers, depending on the experience most needed by a particular student or group. The use of benchmarks (9) and ongoing feedback (1) will also require creative thinking as to what constitutes an effective assessment, drawing on the criteria adapted from Nicol and Macfarlane-Dick (2006) to establish when feedback given is effective and informative and using peer- and video-stimulated approaches to provide *feedback on the feedback*. Finally, adoption into the curriculum (3) will require support not only from students and teachers but programme leaders, facilities managers, and administration. The use of distributed simulation to ensure the *Evaluation Simulator* is as cost-effective and adaptable as possible might help this adoption and lead to lasting change.

### In Research

In its current state, the simulator offers numerous possibilities as a tool for research. By giving controlled, replicable stimuli for evaluation in a heightened setting, it provides a tool to examine the causal relation of environmental and social factors on evaluation procedures. At a fundamental level, studies could be conducted comparing the evaluation of pre-recorded audio and/or video in laboratory conditions (i.e., watching the provided videos on a computer screen) with varying degrees of heightened environmental arousal. Variations could include computer screen only, full-sized projection, or with or without pre-evaluation waiting period, performer stage entrance, or intense lighting. Social features could also be adapted, including informing the participant that the performer is being broadcast live via videoconferencing with possible real-world implications of the evaluation, or by providing additional information about the performer's experience and history.

The variety of pre-programmed responses could be used to examine differences in quantitative and qualitative feedback as affected by the performer's state, including whether a distraught performer triggers empathic reactions and more forgiving evaluations, especially when paired with the good vs. the poor performance. The role of facial features in affecting performance judgment (Waddell and Williamon, 2017b), for example, could be expanded here to see whether a frustrated or distraught reaction following the performance affects how the musical component is remembered and contextualized. In addition to evaluators' written and oral responses, their behavior (e.g., hand gestures, eye contact, rate and pitch of speech, etc.) and physiology (heart, respiratory, skin conductivity, etc.) could be monitored to determine differences across time, especially as they relate to the nature and speed the of feedback given as defined by time to first and final decision described in previous work (Thompson et al., 2007; Waddell and Williamon, 2017b; Waddell et al., 2018).

As the simulator is conducive to panel judgments, it also offers the possibility of examining elements of intra-panel conformity and social response, such as furthering the celebrated conformity studies of Asch (1956), and as examined in music by Radocy (1976). In this manner, one could determine whether artificially positive or negative evaluations from one or more actors playing the role of assumed fellow panelists affect subsequent judgments by the participant. This interaction could be examined at all points of the evaluation: the time spent before the evaluations when “insider” information or initial impressions might be shared; the time during the performance where a variety of non-verbal cues might be used to indicate positive or negative response; direct responses of the actor(s) to the performer; and the time spent after the performer has been dismissed but before the final assessment is provided.

Much remains to be done in understanding the full experience and process of conducting a performance assessment, as well as formalizing approaches to training those performing these crucial judgments. Thus, the intention of the prototype presented here is not to present a fully formed and final approach, but rather to provoke and facilitate the next generation of innovation in performance evaluation understanding and practice.

## Author Contributions

All authors contributed significantly to the conceptualization of the technology described and the preparation of this manuscript.

### Conflict of Interest Statement

The authors declare that the research was conducted in the absence of any commercial or financial relationships that could be construed as a potential conflict of interest.

## Footnotes

^1^For the purposes of this article the terms ‘evaluation’ and ‘assessment’ are used interchangeably and in their educational context, with distinction made between placement, summative, diagnostic, and formative types as defined by Goolsby (1999).

^2^These videos have been compressed as .mp4 files of 1/10 of their original size to allow for their inclusion as Supplementary files. Thus, the audio/video quality is not fully representative of the uncompressed video files as experienced within the simulator.
